# DEVELOPMENT OF AN OPIOID EDUCATION RESOURCE FOR OLDER CANCER PATIENTS

**DOI:** 10.1093/geroni/igz038.2463

**Published:** 2019-11-08

**Authors:** Ruth Manna, Natalie Moryl, Natalie Gangai, Vivek Malhotra, Jennifer Wang, Beatriz Korc-Grodzicki

**Affiliations:** 1 Memorial Sloan Kettering Cancer Center, New York, United States; 2 Memorial Sloan Kettering Cancer Center, New York, New York, United States

## Abstract

Pain is a common problem in older cancer patients, estimated to affect 70% of those with advanced disease. As older adults live longer after diagnosis, the use and misuse of opioids will continue to rise. Gaps in available age-friendly opioid resources for patients were identified at a Comprehensive Cancer Center. An interprofessional team worked to develop a resource to educate older cancer patients and their caregivers regarding safe opioid use. Expert clinical opinions from Supportive Care, Anesthesia Pain, Nursing, and Geriatrics Services as well as patient observations informed drafts of the resource. A total of 22 older patients in three geriatric clinics were approached for a short interview (8 open questions) surrounding opioid understanding and concerns. The most stated concerns were fear of addiction, concern about the opioid epidemic, and potential unwanted side effects. There was an evident lack of awareness of what an opioid was or which one of the medications in their list was an opioid. The interviews underscored the need for the education resource to include names of opioids, address concerns about the opioid epidemic and signs of addiction. Language was added to describe safe use, storage and disposal of opioids. Special considerations in the older adult were emphasized. Links to additional information were provided. Finally, patient education experts reviewed the draft to adapt the language to be patient-friendly. Opioids are often effectively used in cancer pain management and older cancer patients warrant proper education. Patient perspectives are critical in the development of relevant patient education resources.

